# Comparison of SARS-Cov-2 omicron variant with the previously identified SARS-Cov-2 variants in Egypt, 2020–2022: insight into SARS-Cov-2 genome evolution and its impact on epidemiology, clinical picture, disease severity, and mortality

**DOI:** 10.1186/s12879-023-08527-y

**Published:** 2023-08-18

**Authors:** Amr Kandeel, Yassmin Moatasim, Manal Fahim, Hala Bahaaeldin, Rabeh El-Shesheny, Wael H. Roshdy, Mina N. Kamel, Shaymaa Shawky, Mokhtar Gomaa, Amel Naguib, Nancy El Guindy, Ola Deghedy, Reham Kamel, Mohamed Khalifa, Ramy Galal, Mohamed Hassany, Galal Mahmoud, Ahmed Kandeil, Salma Afifi, Amira Mohsen, Mohammad Abdel Fattah, Ghazi Kayali, Mohamed A. Ali, Khaled Abdelghaffar

**Affiliations:** 1 Preventive Sector, Ministry of Health and Population, Cairo, Egypt; 2 Centre of Scientific Excellence for Influenza Viruses, National Research Centre, Dokki, Giza, 12622 Egypt; 3 Department of Epidemiology and Surveillance, Preventive Sector, Ministry of Health and Population, Cairo, Egypt; 4 Central Public Health Laboratory, Ministry of Health and Population, Cairo, Egypt; 5 Public Health Initiatives, Cairo, 11613 Egypt; 6 National Hepatology and Tropical Medicine Research Institute, Ministry of Health and Population, Cairo, 11613 Egypt; 7 Ministry of Health and Population Consultant, Cairo, Egypt; 8 Community Medicine Department, National Research Centre, Cairo, Egypt; 9 Preventive Sector, Central Administration for Preventive Affairs, Ministry of Health and Population, Cairo, Egypt; 10 Human Link, Dubai, United Arab Emirates; 11 Minister of Health and Population, Cairo, Egypt

**Keywords:** SARS-CoV-2 variants, Sequencing, Whole genome, Epidemiology

## Abstract

**Background:**

The o severe acute respiratory coronavirus 2 (SARS-CoV-2) pandemic has killed millions of people and caused widespread concern around the world. Multiple genetic variants of SARS-CoV-2 have been identified as the pandemic continues. Concerns have been raised about high transmissibility and lower vaccine efficacy against omicron. There is an urgent need to better describe how omicron will impact clinical presentation and vaccine efficacy. This study aims at comparing the epidemiologic, clinical, and genomic characteristics of the omicron variant prevalent during the fifth wave with those of other VOCs between May 2020 and April 2022.

**Methods:**

Epidemiological data were obtained from the National Electronic Diseases Surveillance System. Secondary data analysis was performed on all confirmed COVID-19 patients. Descriptive data analysis was performed for demographics and patient outcome and the incidence of COVID-19 was calculated as the proportion of SARS-CoV-2 confirmed patients out of the total population of Egypt. Incidence and characteristics of the omicron cohort from January- April 2022, were compared to those confirmed from May 2020-December 2021. We performed the whole-genome sequencing of SARS-CoV-2 on 1590 specimens using Illumina sequencing to describe the circulation of the virus lineages in Egypt.

**Results:**

A total of 502,629 patients enrolled, including 60,665 (12.1%) reported in the fifth wave. The incidence rate of omicron was significantly lower than the mean of incidences in the previous subperiod (60.1 vs. 86.3/100,000 population, p < 0.001). Symptoms were reported less often in the omicron cohort than in patients with other variants, with omicron having a lower hospitalization rate and overall case fatality rate as well. The omicron cohort tended to stay fewer days at the hospital than did those with other variants. We analyzed sequences of 2433 (1590 in this study and 843 were obtained from GISAID platform) Egyptian SARS-CoV-2 full genomes. The first wave that occurred before the emergence of global variants of concern belonged to the B.1 clade. The second and third waves were associated with C.36. Waves 4 and 5 included B.1.617.2 and BA.1 clades, respectively.

**Conclusions:**

The study indicated that Omicron-infected patients had milder symptoms and were less likely to be hospitalized; however, patients hospitalized with omicron had a more severe course and higher fatality rates than those hospitalized with other variants. Our findings demonstrate the importance of combining epidemiological data and genomic analysis to generate actionable information for public health decision-making.

**Supplementary Information:**

The online version contains supplementary material available at 10.1186/s12879-023-08527-y.

## Introduction

The ongoing severe acute respiratory coronavirus 2 (SARS-CoV-2) pandemic has killed millions of people and caused widespread concern around the world. Multiple genetic variants of SARS-CoV-2 have been identified as the pandemic continues [1]. Analyzing the RNA walk data generated from the SARS-CoV-2 genome provides important information on treatment and vaccine production. Monitoring the circulating variants is crucial as some of them are highly infectious, highly transmissible, resistant to vaccines, and capable of causing more-severe disease.

As of October 2022, a total of 206 countries shared over 6 million genome sequences in the online database of the Global Initiative on Sharing Avian Influenza Data (GISAID) [2]. The international dissemination of SARS-CoV-2 sequences was used for contact tracing and outbreak control, enabling the discovery of variants of concern (VOCs) or other lineages of virological or epidemiological interest [3]. The World Health Organization (WHO) names new coronavirus variants by using Greek alphabet letters; most attention has been focused on the alpha (B.1.1.7), beta (B.1.351), gamma (P.1), delta (B.1.617.2), and omicron (B.1.1.529) variants. Emerging variants that show increased transmissibility and/or immune evasion are classified as VOCs [4].

The omicron variant was first reported in South Africa in October 2021 and has been recognized as a fifth VOC [5]. Within a few months, it became the dominant SARS-CoV-2 strain in South Africa and elsewhere, displacing the delta variant that had led to a devastating surge in cases, hospitalizations, and deaths. Genetic analysis of the omicron variant showed higher mutation rates in the spike protein, representing a distinct evolutionary lineage that deviated from the mainstream of the evolving SARS-CoV-2 detected in mid-2020. The Phylogenetic Assignment of Named Global Outbreak Lineages (Pango Network) [6] separated the B.1.1.529 lineage (omicron) into sister lineages because some related variants lack some mutations identifying their variants [7].

Although less pathogenic than other SARS-CoV-2 VOCs, Omicron’s overall risk remains very high because COVID-19 remains a very high global risk [8]. Furthermore, omicron has higher transmissibility, so it could contribute to the rapid spread of the disease, increase hospitalizations, overwhelm healthcare systems, and lead to higher morbidity, especially in vulnerable groups [9]. In a global risk assessment, WHO has identified four key factors to evaluate Omicron’s overall threat: (i) transmissibility; (ii) effectiveness of vaccination strategy; (iii) virulence of new variant compared to that of other variants; and (iv) the level of understanding, perception, and implementation of control measures, including social and public health measures [10].

In Egypt, the first case of SARS-CoV-2 was announced on 14 March 2020; by the end of May 2022, there have been 513,944 confirmed cases of COVID-19, and 24,718 deaths. Egypt experienced five waves of COVID-19 by the end of May 2022, the last wave starting in the first week of the year and lasting for 16 weeks [11, 12]. By the beginning of the fifth COVID-19 wave, Omicron was the dominant coronavirus variant in Egypt. Vaccination against COVID-19 started 24th January 2021; by the end of May 2022, 46.8% of the Egyptian population were vaccinated with at least one dose, and 34.0% were fully vaccinated.

Concerns have been raised about high transmissibility and lower vaccine efficacy against omicron, with scientists no longer convinced that global vaccination can control COVID-19 on its own. There is an urgent need to better describe how omicron will impact clinical presentation and vaccine efficacy. This study aims at combining epidemiological data and genomic analysis to better describe the epidemiologic, clinical, and genomic characteristics of the omicron variant compared to other VOCs.

## Patients and methods

### Study design and setting

We have performed a secondary data analysis using the results from Egypt’s National Electronic Disease Surveillance System (NEDSS), which was established in 2002. NEDSS is a laboratory-based surveillance program targeting 41 infectious diseases through an online, web-based application. Altogether, 284 governmental hospitals, including chest, general, and infectious diseases hospitals, and more than 5,300 primary health units throughout the country served as reporting sites. Data of all COVID-19 confirmed patients seen at all health facilities reporting to NEDSS were used. Because nearly three-quarters of Egypt’s healthcare services are provided by the public sector [13], other hospitals of the public sector, including teaching, health insurance, and university hospitals, were invited to voluntarily report aggregate NEDSS data on acute respiratory infection (ARI) to the Ministry of Health and Population (MoHP) each week. All patients attending outpatient clinics or admitted to governmental hospitals are reported through NEDSS within 48 h; SARS-CoV-2 was included in the ARI testing panel in 2020.

### Target population and data collection

The subjects are all patients with ARI who were seen at the outpatient clinics or hospitalized with a history or measured fever of ≥ 38 °C and cough within the 10 days before disease onset. Enrolled patients were interviewed by the hospital surveillance officers using the standard surveillance form that includes the patient’s demographic data, signs and symptoms, and ARI risk factors. Data were entered by using the online NEDSS application. All patients were requested to provide oropharyngeal and nasopharyngeal swabs for SARS-CoV-2 testing by RT-PCR at the nearest regional laboratory or Central Public Health Laboratory in Cairo.

### Study period

The total study period (May 2020 – April 2022) was subdivided into four-month subperiods (May-August 2020, September-December 2020, January-April 2021, May-August 2021, September-December 2021, January-April 2022), with omicron predominated between January and April 2022 (omicron cohort).

### Sample collection and processing

Oropharyngeal or nasopharyngeal swabs were collected from patients at Central Public Health Laboratories at MoHP with suspected SARS-COV-2 infections from March 2020 to 14 May 2022. Swab samples were collected on DMEM media supplemented with 2% BSA and 2% antibiotic antimycotic. After being subjected to genetic material extraction using a KingFisher® Flex extraction machine (Thermo Scientific), SARS-CoV-2 was detected by performing real-time RT-PCR using N gene and ORF1ab primers and probes using a VIASURE SARS-CoV-2 RT-PCR Detection Kit (Certest Biotec SL, Spain).

### SARS-CoV-2 whole-genome sequencing

First, cDNA strands for the viral genome of each sample were synthesized, and double strands were amplified by using the SuperScript™ IV One-Step RT-PCR System. After PCR purification and cleanup, the Illumina Nextera XT DNA library prep kit for MiniSeq illumina Sequencing System was used. CLC Genomics Workbench version 20 (CLC Bio, Qiagen) workflow was then used to align the reads with the reference genome (NC_045512.2). NGS was performed at the Center of Scientific Excellence for Influenza Viruses laboratory at the National Research Centre, Egypt.

### Sequence alignment and phylogenetic analysis

Full Egyptian viral genomes and metadata are available on the GISAID initiative (EpiCoVTM) platform. Full genomes of SARS-Cov-2 viruses from Egyptian patients were downloaded from the GISAID initiative (EpiCoVTM) database on 14/5/2022 (843 sequences from institutions other than National Research Center and Central Public Health Laboratory, Egypt). A total of 2,433 virus genomes were submitted to https://clades.nextstrain.org/ V1.14.0 [10] for the classification of lineages and sub-lineages; 27 sequences failed the analysis, and 2406 succeeded.

### Data analysis methods

We conducted a retrospective analysis of the NEDSS data during the COVID-19 pandemic in Egypt. The data of COVID-19-confirmed cases between March 2020 and April 2022 was obtained. All patients with ARIs who attended as outpatients or were admitted to MoHP hospitals during this period were included. Surveillance officers at each reporting site regularly checked data for completeness and validity using facility records. Descriptive data analysis was performed for demographics, history of comorbidities, and patient outcome by using Epi info7. The incidence rate of COVID-19 during each subperiod was calculated by determining the proportion of SARS-CoV-2 confirmed patients out of the total population of Egypt. Incidence in the omicron period was compared to that during the previous subperiods. Characteristics of the omicron cohort from January- April 2022, were compared to those of the patients who presented to MoHP hospitals from May 2020 to December 2021 by using bivariate analysis. Comparison variables included age, sex, days from symptom onset to hospitalization, chronic conditions, year, season, region, and SARS-CoV-2 genotypes. Pearson’s chi-square was used to evaluate the difference between categorical variables, the t-test was used for continuous variables, with statistical significance set at P value < 0.05.

Characteristics of patients who died of omicron were compared with those of patients who died before the emergence of omicron to examine the differences in mortality risk between omicron and other SARS-CoV-2 variants.

## Results

### Phylogenetic analysis of SARS-CoV-2 genomes

**Fig. 1:**
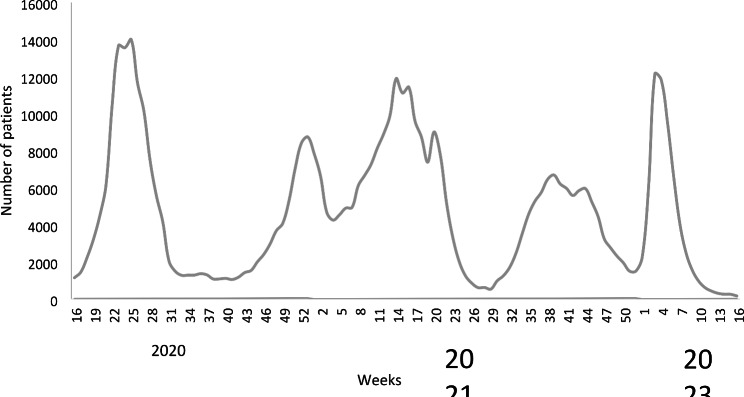
Distribution of COVID-19 over time in Egypt 2020–2022

According to the genomic analysis by https://clades.nextstrain.org/, 96 different Pango lineages were detected in Egypt during the study period. Data of COVID-19 confirmed cases between March 2020 and April 2022 show that five SARS-CoV-2 waves were recorded in Egypt based on epidemiological data (Fig. 1). The most prevalent variants in the first wave were B.1 (53%), followed by C.36 (20%), then B.1.1 (9.98%), and other B variants (9%). During the second wave, the most prevalent variant was C.36 (46%), followed by B.1 (32%), and other B variants (14.09%), then C.32 (2.2%). During the third wave, C.36 was the most dominant variant (42%), followed by C.36.3 (29%), then B.1 (4.8%), B (%3.5), A.28 (3.3%), and C.38 (2.8%).

B.1.617.2 (40%) was the most dominant variant during the fourth wave, followed by AY.122 and AY.124 (11% each), then AY.127 (3%), B.1 (3.9%), AY.20 (3%), then AY.43 and AY.45 ( 2.5% each). The fifth wave showed the highest prevalence in omicron strains BA.1 (32%), BA.1.1 (19%), BA.1.18 (6%), and BA.2 (14%), followed by B.1.617.2 (9%), AY.122, AY.124 (1.7% each).

**Fig. 2:**
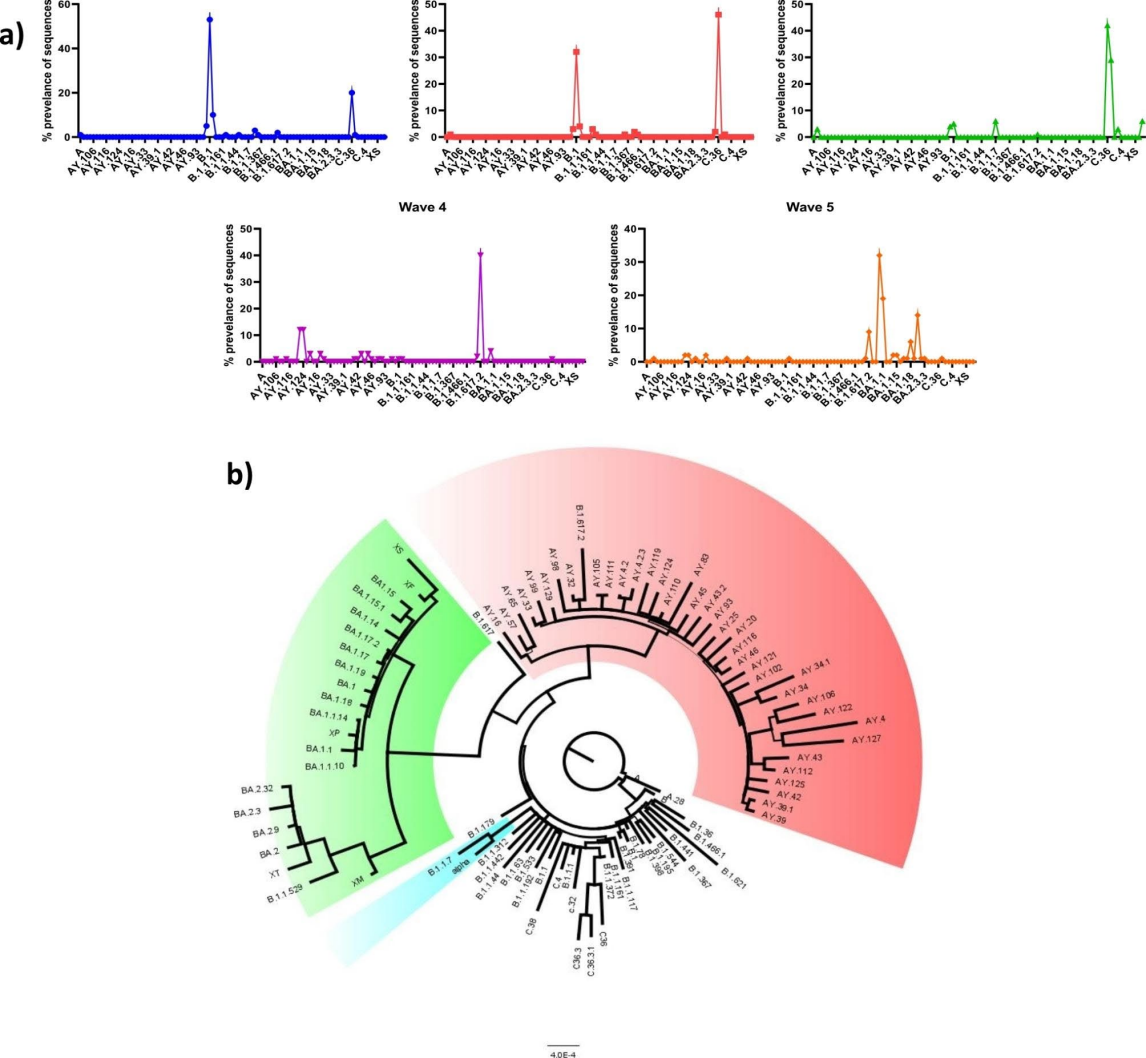
detection of SARS-CoV-2 variants during each of the 5 waves (**A**). Circular phylogenetic tree depicting evolutionary relationship of Egyptian SARS-CoV-2 sequences (**B**)

The phylogenetic tree of the viruses detected in Egypt indicates that delta variants showed greater genetic diversity (39 different Pango lineages) followed by omicron (21 different Pango lineages) (Fig. 2).

### Distribution of SARS-CoV-2 lineages and clades

**Fig. 3:**
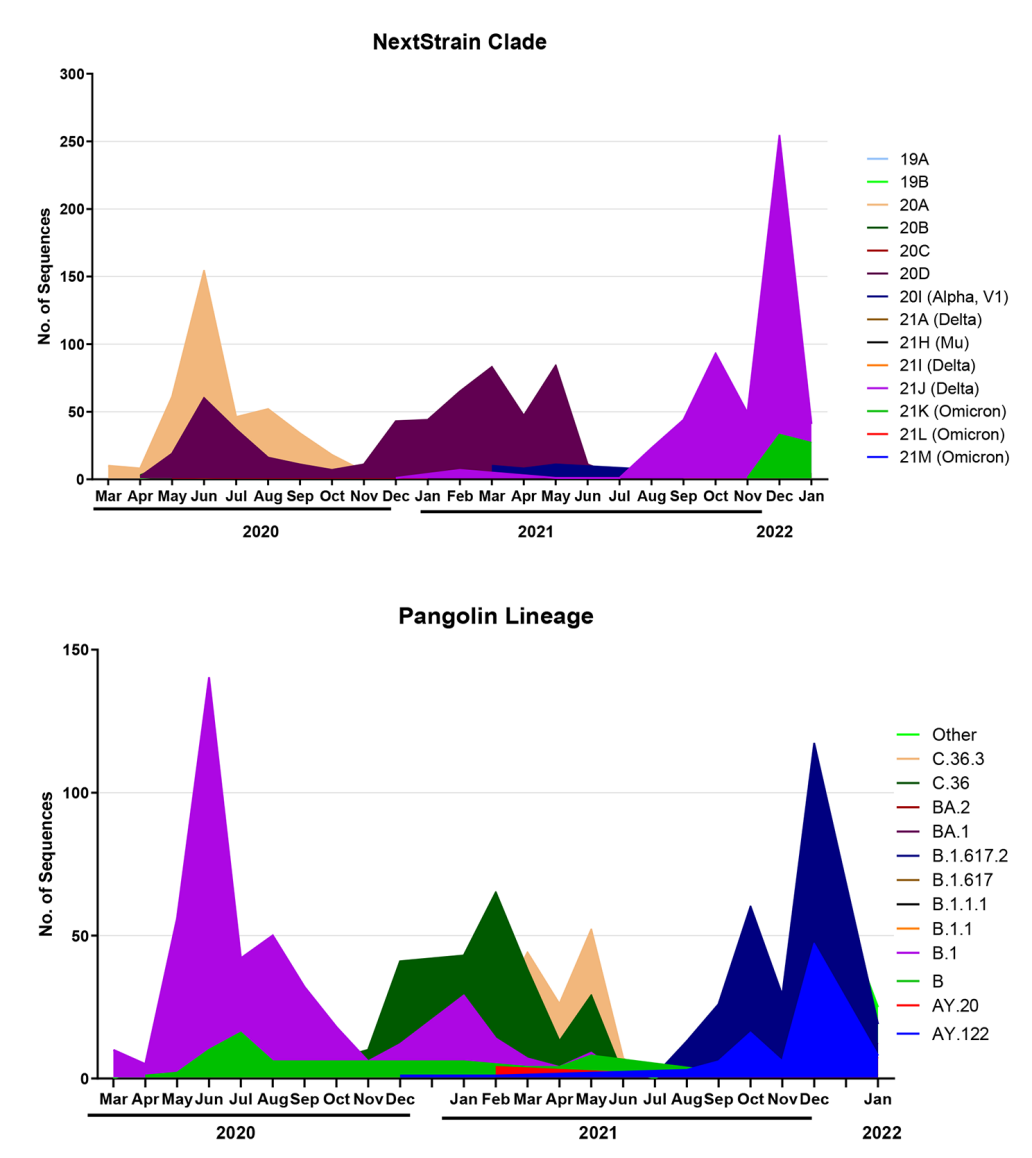
Prevalence rates of SARS-CoV-2 NextStrain and Pangolin lineages and sublineages in Egyptian isolates

Although omicron sequences represented 20.95%, delta formed 24.95% of all published sequences and was divided into 3 clades: 21 A (1.3%), 21I (1.7%), and 21 J (97%). 21 A (Delta) harbors (AY.16 and B.1.617.2). 21I (Delta) harbors (AY.57, AY.65, and B.1.617.2). 21 J (Delta) harbors various AY lineages and B.1.617.2. Delta B.1.617.2 VOC was first detected in India in October 2020, and in Egypt in February 2021. Delta VOC Pango lineage B.1.617.2 represents 20% of 21I, 52.8% of 21 J, 89% of 21 A, and 52.7% of all delta variant samples in Egypt (Fig. 3).

Alpha clade 20I lineage B.1.1.7 was first identified in the United Kingdom in September 2020 and identified as a variant of concern by WHO in December 2020. All Egyptian alpha sequences are classified as (20I) linage, B.1.1.7 clade. Alpha sequences represent (13.7%) of all published Egyptian sequences. Most of these samples were collected from April to May 2021. Two more samples were detected by the Egyptian Ministry of Health in December 2021.

20D clade represents 23.6% of all sequences and harbors the following Pango lineages: C.36 (68%), C.36.3 (27.6%), C.38 (2.65%), C.32 (1.6%), B.1.1.1 (0.53%), and C.4 (0.18%) of all 20D sequences. Although B.1.1.1 and C.32 were not detected in Egypt in 2021 and C.36.3.1, C.38, and C.4 were last detected in June 2021, C.36 and C.36.6 continued circulating in Egypt and were detected in December 2021, and January 2022, respectively.

20B clade forms 4.9% of all Egyptian sequences and is mainly represented by Pango lineage B.1.1, forming 70.3% of 20B sequences, and is still circulating in Egypt with low frequency. 20 A was recently detected in Egypt and is circulating at a low frequency. 20 A sequences represent 20.7% of all SARS-CoV-2 sequences from Egypt, represented mainly by B.1 Pango lineage (88.6%).

### Analysis of omicron genome sequences

Omicron sequences represented 20.95% of all sequences obtained from Egypt. NextClade/ strain analysis showed that Egyptian omicron strains are divided into 3 clades: 21 K (82.1%), 21 L (13.9%), and 21 M (4%). Additionally, 21 K is further split into BA.1 (represented by 51.3% of the sequences), BA.1.1 (27.6%), BA1.14 (4.35%), BA.1.15 (2.66%), BA.1.17 and BA1.17.2 (4.1%), BA.1.18 (9%). and BA.1.19 (2%). The omicron VOC (B.1.1.529 Pango) is represented by only two sequences in Egypt, and the monitored amino acid change S:346 K is present in BA.1.1, representing 25% in all omicron samples. 452R and 486 V mutations are not present in Egypt.

### Genomic variation and mutation signature

#### Spike gene

**Fig. 4:**
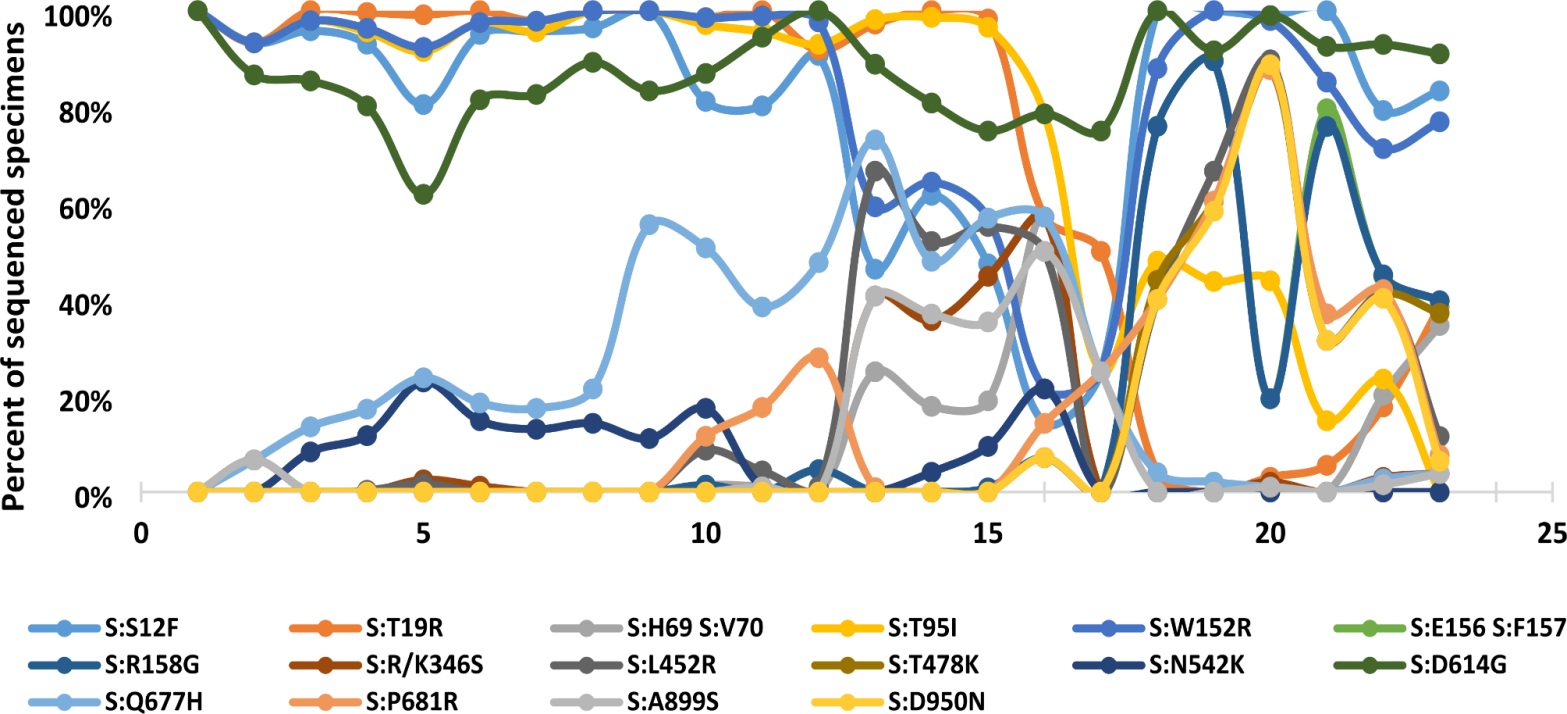
variations in all Egyptian strains sequences of SARS-CoV-2 from Wuhan-1 strain

Whole-genome sequences obtained from Egyptian SARS CoV-2 viruses were compared with the Wuhan-Hu-4 reference sequence (GISAID: EPI_ISL_402124) to detect mutations in the Spike gene (Fig. 4). Analysis of the spike (S) glycoproteins of Egyptian SARS CoV-2 viruses indicated that 94.9% of all Egyptian sequences have D614G forms. Also, data showed that despite being less frequent, viruses harboring D614 are still detected among Egyptian variants. Egyptian omicron strains belong to 3 clades: 21 K, 21 L, and 21 M. The spike protein of 21 K and 21 L clades share the following substitutions: 339D (80.7%), 371 L/F (82.5%), 375 F (81.8%), 417 N (82.6%), and 440 K (78.6%) in S1 RBD, 655Y (94.7), and 679 K (98.2%) in the SD1/2 subdomains of S1, 681 H (98.2%), 764 K (94%), 796Y (98% ), 954 H ( 98.4%), and 969 K (96.5%) in the S2.

The 21 K omicron samples significantly and specifically showed S1: H69-V70 deletion in 93%, T95I in 100% of 21 K, Y145D in 91.5%, N211 deletion and l212I in 92.6% in the spike N terminal domain (NTD) of the Egyptian omicron samples, 371 L (82.5%), and 346 K (25% of all Egyptian omicron samples) only in RBD of BA.1.1 clade samples, and 547 K in 93.2% of all samples. The 21 K samples share significant S2 mutations, 856 K in 93.8% and 981 F in 93%.

All 21 L omicron samples shared the following mutations (not present in 21k): 19I, 24:26 deletion, 27 S, 142D, and 213G in S1(NTD), and (376 A, 405 N, and 408 S) in the RBD.

Delta (21 A, 21I, and 21J1) clades shared various significant mutations in the spike protein; D614G was present in 98.4 of all delta sequences. S: T19R(97.7%), S: T478K (96.1%), S: P681R (96%), S: L452R (95.5%), S: E156 S: F157 deletion (76.4%), S: R158G (76.3%), S: D950N (52%), and S: T95I (50.6%). WHO monitored mutation: K417N was not present in any sequenced sample in Egypt, and S: E484K was present in 1.6% of the samples, (all belong to B.1.617.2 Pango lineage in the 21 J next-strain clade).

Other substitutions were present in a few samples, such as G142D (15.3%) and G142-deletion in 3.4% of all delta sequences and 5.6% of B.1.617.2 Pango clade. Other substitution (A178H) forms (3.7%) of all delta samples and is only present in VOC B.1.617.2 Pango clade represented by (7.44%) of this clade. S: H69 S: V70 deletion is present in 8.7%, T572I in 5.1%, S254F in 2.4%, and P209S in 1.9% of all delta sequences.

Alpha WHO clade (20I), B.1.1.7 Pango lineage sequences all harbor the A570D and S: D614G substitutions. Other substitutions are considered dominant in the spike protein: S: P681H (97%), T716I (97%), N501Y (94%), D1118H (94%), and S982A (63%) of all alpha sequences. S1: H69-V70 deletion is present in 25% of Egyptian alpha sequences.

20D clade is also designated by S: D614G and S: Q677H substitutions, represented in 98% and 81% of the sequences. Although S12F and L452 substitutions are present in 40% of 20D samples and A899S is in 26%, these substitutions are more dominant in 20D C.36.3 Pango lineage, representing 90%, 80%, and 86% of this lineage, respectively. Other substitutions are restricted to C.36.3 and C.36.3.1 lineages in the 20D clade: R346S (95%), W152R (90%), H69-V70- deletion (51%), and less-frequent substitutions, such as A871S (8%) and V120I (5%) of C.36.3 sequences. Other substitutions are restricted to C.36 lineage: P681R (13%), V1264L (9%), S477N (5%), and P384L (2%). Others were predominantly present in C.38: E484K (92%), W64R (71%), C1243S(33%), D138Y (29%), D138-(14%), and V1264M (25%) as percentages of the total number of sequences in each lineage.

20B clade sequences exhibited 3 substitutions: S: D614G (97.6%), A1078S (16%), and L5F (9%). 20 A also showed D614G substitution in (99.7%), and E554D in (6.5%) of its sequences. D614G substitution was not detected in any 19B sample sequences and in only 14% of 19 A clade sequences.

#### Other genes of SARS-CoV-2

For N-gene several substitutions are shared among different lineages and clades: R203K (omicron, alpha, 20D, and 20B) or 203 M (delta and 20 A), G204R (omicron, alpha, 20D, and 20B), G215C (delta and 20 A), and P13L (omicron and 20 A), are present in 47, 42, 26, 22, and 18% of all Egyptian SARS-CoV-2 samples. Omicron sequences showed P13L in (94%), E31- R32- S33- deletion in 97%, R203K in 97%, G204R in 98%, and S413R in 17% (in 97% of 21 L sequences). Omicron sequences showed D63G in 92%, R203M in 98%, D377Y in 97%, G215C in 96%, and S232N in 2%. 20I Alpha sequences showed D3L, R203K, G204R, and S235F in all sequences. 20D sequences showed R203K in 96% of the 20D corresponding sequences, G204R in 94%, G212V in 93%, S193I in 7% (restricted to C.36), T366I in 6% (restricted to C.36), E378Q in 3% (restricted to C.38), and Q390K in 2% of 20D sequences (restricted to C.36.3 sequences). R203K and G204R are present in all 20B sequences. S202N is present in all 19B sequences, with A35V in 78% of them. 20 A samples represent S235F (19%), A35V (8%), P13L in (5%), M234I in (7%), R203M and G215C in (3%) of 20 A samples (Fig. 4).

For the E-gene we show that T9I substitution is present in all omicron sequences; D72Y is represented in some 20D (C36.3) samples, and V5F is in some 20 A. Three main substitutions are present in the M-gene of omicron sequences: A63T in 96%, Q19E in 87% of all omicron sequences, and D3G in 49% of 21 K clade sequences. I82T substitution is present in 92% of 21 J (delta) sequences and 94% of C.36.3 (20D) sequences; T175M was detected in 3% of C.36.3 (20D) sequences. Our analyses showed that other genes of SARS-CoV2 had several mutations (Table S1).

### Comparison of omicron and previous SARS-CoV-2 variants

#### Incidence rate and demographic characteristics of COVID-19

**Fig. 5:**
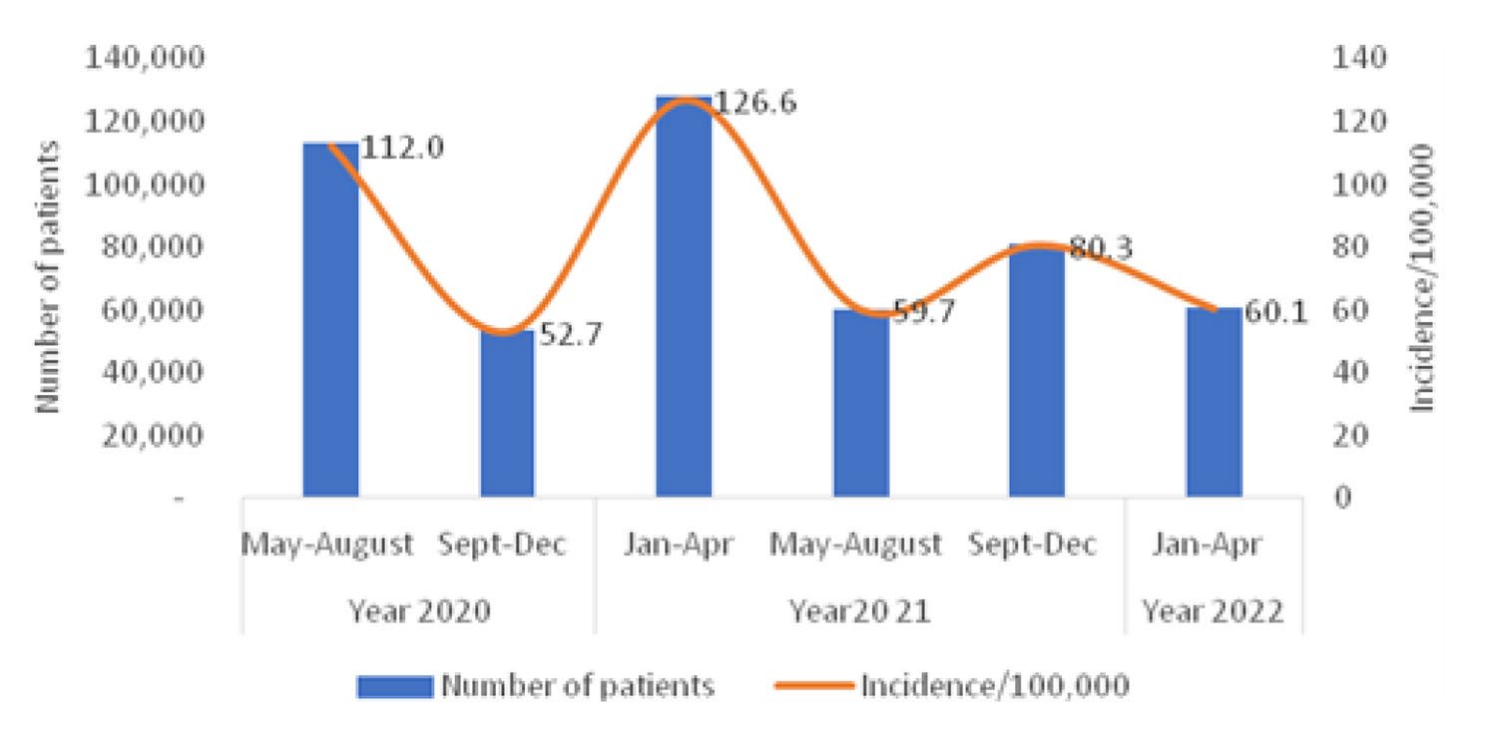
Number of cases and incidence of COVID-19 in the omicron period compared to previous periods, Egypt 2020–2022

Between March 2020 and May 2022, a total of 502,629 patients were confirmed to have COVID-19 infections. There were five distinct waves of COVID-19 identified in Egypt over who, the fifth of which occurred between January and April 2022, with 60,665 (12.1%) cases reported mainly due to the omicron variant (omicron cohort) (Fig. 1). The incidence rate of omicron was significantly lower than the mean of incidences in the previous subperiod (60.1 vs. 86.3/100,000 population, p < 0.001) (Fig. 5).

Omicron tended to infect younger age groups and female patients more than other variants did. The percentages of patients infected with omicron were significantly higher in age groups 1–15 y and 16–50 y (2.6 and 57.3% vs. 2.2 and 52.7%, p < 0.001 respectively), females (52.7 vs. 50.2%, p < 0.001) and residents of the urban and frontier governorates (50.2 and 3.3% vs. 46.4 and 2.6%, p < 0.001) compared to other types of variants. Patients with omicron were less likely to have comorbidities and seek healthcare after two days from disease onset than were those with other variants (18.8 vs. 21.0%, p < 0.001 and 30.5 vs. 35.4%, p < 0.001 respectively) (Table 1).

**Table 1:** Characteristics of patients infected with SARS-CoV-2 Omicron compared to other variants

Characteristics	Omicron (N = 60,665)		Other variants (N = 441,964)		OR	95% CI	P value
	Number	Percent	Number	Percent			
Age group							
1–15	1,573	2.6%	9,664	2.2%	1.2	1.13–1.26	< 0.001
16–50	34,781	57.3%	232,966	52.7%	1.2	1.19–1.23	< 0.001
51–65	12,306	20.3%	118,523	26.8%	0.7	0.68–0.71	< 0.001
> 65	12,005	19.8%	80,811	18.3%	1.1	1.10–1.13	< 0.001
Gender							
Male	28,671	47.3%	219,885	49.8%	0.9	0.89–0.92	< 0.001
Female	31,994	52.7%	222,027	50.2%			
Missing	0		52	0.0%			
Region							
Urban	30,446	50.2%	205,768	46.6%	1.2	1.14–1.18	< 0.001
Upper Egypt	19,019	31.4%	146,977	33.3%	0.9	0.90–0.93	< 0.001
Lower Egypt	9,176	15.1%	77,943	17.6%	0.8	0.81–0.85	< 0.001
Frontier	2,024	3.3%	11,276	2.6%	1.3	1.26–1.38	< 0.001
Duration onset – seeking healthcare							
0–2	25,854	42.6%	162,098	36.7%	1.3	1.26–1.31	< 0.001
3–10	17,406	28.7%	147,090	33.3%	0.8	0.79–0.82	< 0.001
> 10	1,084	1.8%	9,289	2.1%	0.8	0.79–0.90	< 0.001
Missing	16,321	26.9%	123,487	27.9%	0.9	0.93–0.97	< 0.001
Comorbidities	11,385	18.8%	92,754	21.0%	0.9	0.85–0.89	< 0.001
Symptoms							
Fever	34,223	56.4%	277,232	62.7%	0.8	0.76–0.78	< 0.001
Dyspnea	18,278	30.1%	161,767	36.6%	0.7	0.73–0.76	< 0.001
Diarrhea	5,115	8.4%	55,595	12.6%	0.6	0.62–0.66	< 0.001
Pneumonia	8,232	13.6%	90,784	20.5%	0.6	0.58–0.62	< 0.001
Case fatality rate	3,884	6.4%	34,438	7.8%	0.8	0.78–0.84	< 0.001
Hospitalized	13,395	22.1%	145,237	32.9%	0.6	0.57–0.59	< 0.001
Hospital stay (days)							
0–7	7,536	56.3%	68,665	47.3%	1.4	1.38–1.49	< 0.001
8–14	2,237	16.7%	26,135	18.0%	0.9	0.87–0.96	< 0.001
> 14	906	6.8%	11,166	7.7%	0.9	0.81–0.93	< 0.001
Missing	2,716	20.3%	39,271	27.0%	0.7	0.66–0.72	< 0.001
ICU admission	2,108	15.7%	13,656	9.4%	1.8	1.71–1.89	< 0.001
Ventilation	1,154	8.6%	9,051	6.2%	1.4	1.33–1.51	< 0.001
Died at hospital	2,775	20.7%	24,005	16.5%	1.3	1.26–1.38	< 0.001

Different symptoms, including fever, dyspnea, diarrhea, and pneumonia, were reported less often in the omicron cohort than in patients with other variants, with omicron having a lower hospitalization rate and overall case fatality rate as well.

The omicron cohort tended to stay fewer days at the hospital than did those with other variants; however, when hospital admission is needed, omicron could have a more severe disease course in terms of ICU admission, ventilation, and death at the hospital than other variants. (Table 1).

#### The relative risk of death from omicron compared to other variants

The mortality rate of the omicron cohort was significantly lower than that of patients with other variants (6.4 vs. 7.8%, p < 0.001). Omicron fatalities occurred more in the two extremes of age (< 15 years = 1.0 vs. 0.5% and > 65 years = 68.3 vs. 51.4%) and in males, patients with comorbidities, hospitalized patients, those admitted to hospital within 2 days of symptom onset, those who stayed < 7 days at the hospital, those admitted to the ICU, and those who required mechanical ventilation (Table 2).

**Table 2:** Risk of mortality from omicron compared to risk from previous circulating SARS-CoV-2 variants, Egypt March 2020-April 2022

Characteristic	Omicron (N = 3,884)		Other variants (N = 34,438)		OR	95% CI	P value
	No.	Percent	No.	Percent			
Case fatality rate	3884	6.4%	34,438	7.8%	0.8	0.78–0.84	< 0.001
Age group (years)							
1—15	39	1.0%	157	0.5%	2.2	1.56–3.15	< 0.001
16–50	285	7.3%	4475	13.0%	0.5	0.47–0.60	< 0.001
51–65	908	23.4%	12,093	35.1%	0.6	0.53–0.61	< 0.001
> 65	2652	68.3%	17,713	51.4%	2.0	1.89–2.18	< 0.001
Male sex	1996	51.4%	17,105	49.7%	1.1	1.00-1.14	0.042
Region							
Lower Egypt	1552	40.0%	11,466	33.3%	1.3	1.25–1.43	< 0.001
Upper Egypt	1124	28.9%	12,009	34.9%	0.8	0.71–0.82	< 0.001
Urban	1055	27.2%	10,064	29.2%	0.9	0.84–0.97	0.007
Frontier	153	3.9%	899	2.6%	1.5	1.281.82	< 0.001
Comorbidity	1719	44.3%	14,206	41.3%	1.1	1.06–1.21	< 0.01
Hospitalized	2775	71.5%	24,005	69.7%	1.1	1.01–1.17	0.026
Onset - admission							
0–2	1165	42.0%	9518	39.7%	1.1	1.01–1.19	0.019
3–10	1477	53.2%	12,330	51.4%	1.1	1.00-1.17	0.070
> 10	63	2.3%	625	2.6%	0.9	0.67–1.13	0.323
Missing	70	2.5%	1532	6.4%	0.4	0.30–0.48	< 0.001
Hospital stay (days)							
0–7	1607	57.9%	12,482	52.0%	1.3	1.17–1.38	< 0.001
8–14	638	23.0%	5168	21.5%	1.1	1.00-1.20	0.08
> 14	328	11.8%	2675	11.1%	1.1	0.95–1.21	0.229
Missing	202	7.3%	3680	15.3%	0.4	0.37–0.50	< 0.001
ICU admitted	839	21.6%	5428	15.8%	1.5	1.36–1.60	< 0.001
Ventilated	471	12.1%	3088	9.0%	1.4	1.26–1.55	< 0.001

## Discussion

We analyzed the genomic epidemiology of SARS-CoV-2 in Egypt during 2020–2022, detecting approximately 96 unique viral lineages. Our data show that the virus was introduced into Egypt multiple times. Genomic surveillance showed that the dynamics of the SARS-CoV-2 lineages circulating in Egypt are associated with the introduction of new VOCs, as recorded in the fourth (delta) and fifth (omicron) waves. B.1 was the most dominant variant in the first wave, and C.36 was largely detected during the second and third waves. Our analysis showed that the beta variant was not detected in Egypt, and the number of alpha variants was limited. Previous studies showed that the prevalence of both C.36 lineages with L452R substitutions and 69–70 del substitutions was high in Egypt at the time of alpha and beta variants [14–16] indicated that C.36 lineages were predominant and acquired several mutations known to confer an adaptive advantage.

The dynamics of SARS-CoV-2 spreading in Egypt were similar to those reported globally. With first and second waves dominated by viruses belonging to B.1 and C.36 lineages, followed by a third wave linked to the circulation of C.36 lineage that acquired several mutations in spike protein and evolved into sub-lineages. We show that the delta variants had greater genetic diversity, with 39 different Pango lineages, followed by omicron, with 21 different Pango lineages and most dominant variant during the fourth wave and replacing previously circulating variants, the delta variant had additional mutations hat contributed to its increased transmissibility and rapid spread worldwide [17–19].Studies reported that the evolution of SARS-CoV-2 to the omicron variant has resulted in mutations conferring a more-contagious nature and vaccine escape [20–22]. Many of the mutations in the spike protein of omicron could impair the antibodies’ ability to bind to the virus, reducing the effectiveness of a vaccine or prior infection at preventing new infections. However, researchers were unable to determine whether omicron is less pathogenic than earlier variants because of preexisting acquired or natural immunity [23]. This study reported a reduction in SARS-CoV-2 incidence during the omicron wave compared to the previous waves. The reduction in incidence is likely to be due to many factors, including the change in health-seeking behavior, changes in triage procedures as the pandemic progressed, omicron’s mild symptoms, decreased fear of the disease, an increase in vaccine-induced immunity in the population, and a preference for laboratory testing and hospitalization in moderate to severe cases. Other reasons may include the high pre-vaccination humoral immunity levels identified among the Egyptian population and the cross-protective immunity between omicron and delta variants [24].

This study reported an increase in the percentage of infections within the urban and frontier governorates regions. Other studies also reported different infection rates of omicron by geographic regions [5]. The reasons for omicron’s increased transmissibility in urban areas could be the high population density, whereas the inaccessibility of healthcare services, low vaccination coverage, and low level of awareness and healthcare-seeking could be reasons in the front.

Earlier studies have reported omicron to be less severe than the predecessor variants. A study by Wolter et al. conducted in South Africa found that omicron hospitalization is 80% lower than that caused by other SARS-CoV-2 variants [25]. Following this, our study identified a lower hospitalization rate among the omicron cohort, possibly due to the scarcity of mutations in the omicron genes that the T cells target, leading to the preservation of prior immunity acquired from previous infections [26].

Studies also reported lower rates of admission to intensive care, need for mechanical ventilation, and death at hospitals among omicron patients for the same reasons [27, 28]. In contrast to this, we found higher rates of ICU admission, mechanical ventilation, and death at hospitals in omicron patients, even with the lower incidence, milder symptoms, and significant reduction in overall mortality rates attributed to omicron compared to previous variants. The higher ICU and at-hospital deaths could be related to the selective admission of the more-severe cases because of the higher rates of asymptomatic and mild cases. This assumption could be supported by the shorter time between onset and admission and the shorter length of hospital stay, indicating the severity of cases.

We found that omicron infects younger patients and females more than other variants do, following the findings of other studies [23, 29].

The lower case fatality rate of omicron compared to that of other variants noted in this and other studies could be explained by the ability of omicron to replicate in the upper respiratory tract more than the lungs, leading to a reduced risk of death [30].

The mortality rate of omicron in this study was higher among males and extremes of age, findings also reported by other studies [27, 30–32]. The risk of death from omicron infection was higher among hospitalized patients with higher comorbidities, those who were admitted to the ICU, and those requiring mechanical ventilation than other SARS-CoV-2 variants were. This discrepancy could be explained by the selective admission of the more severe cases in Egypt as the pandemic progresses. Other factors that could be related to the omicron higher ICU, ventilation, and at-hospital mortality rates could include comorbidity, a previous COVID vaccine-induced immunity, and COVID natural immunity [33].

## Conclusions

By tracking the prevalence of different variants of SARS-CoV-2 among Egyptians from February 2020 to June 2022, we found lower incidence, milder disease symptoms, and lower mortality among patients infected with omicron than in those infected with other SARS-CoV-2 variants. Different demographic and epidemiologic characteristics of omicron were identified and compared to those of previous variants. Omicron-infected patients had milder symptoms and were less likely to be hospitalized; however, patients hospitalized with omicron had a more severe course and higher fatality rates than those hospitalized with other variants.

A more-robust genomic strategy of surveillance is needed to better describe genomic changes of SARS-CoV-2 across the country at regular time intervals. Although sequencing efforts in Egypt and other countries have improved, the number of sequences remains quite low compared with the number of recorded cases. Enhanced genomic surveillance for SARS-CoV-2 connected to epidemiologic data in Egypt can support the early detection of emerging variants and assist with improving control strategies.

### Study limitations

The number of obtained sequences was not equally distributed for different waves. Another study limitation is that the data were from MoHP hospitals, which represent only 1/3 of the healthcare system in Egypt. Additionally, due to the difficulty in defining the duration of each wave separately, we were unable to compare the fifth wave period to previous waves. During each wave, a mixture of VOCs was identified. Starting in January 2022, however, the dominance of the omicron variant defined the beginning of the fifth wave. Lastly, there may be other factors affecting the difference in incidence besides variant infectivity, such as previously acquired infections or vaccinations; however, these variables were not studied.

### Electronic supplementary material

Below is the link to the electronic supplementary material.

Supplementary Material 1

## Data Availability

All data used in this study are available online through publicly open databases (10.55876/gis8.230312uh). SARS-CoV-2 genomes for Egypt were retrieved from the global initiative on sharing all influenza data (GISAID) database (https://www.gisaid.org/).
