# Supplementary material for: Comparison of SARS-Cov-2 omicron variant with the previously identified SARS-Cov-2 variants in Egypt, 2020–2022: insight into SARS-Cov-2 genome evolution and its impact on epidemiology, clinical picture, disease severity, and mortality
Source: BMC Infect Dis. 2023 Aug 18;23:542. doi: 10.1186/s12879-023-08527-y (PMC10439637; doi:10.1186/s12879-023-08527-y)
Supplement: Supplementary file 1 — Supplementary Material 1 [file 12879_2023_8527_MOESM1_ESM.docx]

Table S1

Mutations in other genes of SARS-CoV-2 in all Egyptian strains sequences of SARS-CoV-2 from Wuhan-1 strain

| **Gene** | **OMICRON** | **DELTA** | **20D** | **Alpha 20I** | **OTHER** |
| --- | --- | --- | --- | --- | --- |
| **ORF1a** | T3255I (97% of all delta and omicron ) | | H83Y (5%), P361L (3%), A3615V (2%), R207C (2%), G3334S (2%), and T265I (2%).   T1246I ( 98% 20D) , G3278S (87% 20D), T4090I (65% 20D)  A859V (49% 20D), L3691S (44% 20D), D2980N (44% 20D), D3222N (44% 20D), D1639N (39% 20D), P2287S (39% 20D), and E102K (31% 20D) | T1001I and I2230 in all sequences    A1708D in 97%,  K2200N in 35%,  and G519S in 23% | A3529T in 79% (19B), V86F in 79% (19B), G2118C in 38% (19B), and K3353R in 38% (19B) |
|  | P3395H (93% 21K and 21L )  **21K**  S2083- (100% 21K) , 2084I (100% 21K) , L3674- S3675- G3676- deletion (90% 21K), I3758V (98% 21K), K856R (96% 21K), and A2710T (98% 21K)  **21L**  G1307S (100% 21L), T3090I (97% 21L), T842I (92% 21L), L3201F (69% 21L), S3675-, G3676-, F3677- deletion (97% 21L) | A1306S (88%) G3334S (9%), and V3660M (3%) of all delta  T265I (42.9% of 21I) and (15.5% of 21J), R207C (16% 21J) and (50% 21I)  **21J**  T3255I (95% 21J ), P2046L (93%21J ), T3646A (91% 21J ), P2287S (90% 21J ), and V2930L (75% 21J ), K669N (7% 21J ) and R1464W (6% 21J ).  **21I**  3209V (90% 21I) , P1640L (88% 21I ) |  |  |  |
| **ORF1b** | P314L substitution was detected in 92% of all Egyptian sequences | | | | |
|  | 1566V (87%), R1315C (17%), and T2163I (20%) | G662S (84%), P1000L (84%), A1918V (76%), V972I(21%) ,and N1030Y (14%) | A1219S, N1426Y, and M1693I (>20% C.36 ), |  | A975V (69% 19B) and D1183Y (89% 19B) , V1092I and S1159T (>10% 20B) |
| **ORF7a** |  | T120I and V82A (>90% of all Delta) , P45L (47% AY.122) and R118G (33% of AY.122) |  |  |  |
| **ORF7b** | T40B (27%) | T40B (97%) | T40B (31%) |  |  |
| **ORF6** | D61L (99% of 21L) |  |  |  |  |
| **ORF8** |  | D119- F120- deletion (85% ) | T11K in all C.38 | Q27* & K68* stop codon immature termination (97%) , Y73C in all, and R52I in (58%) | L84S (97% 19B) |
| **ORF3** | T223I (96% OF 21L) | S26L (96.6%) | S171L (10.4% C.36) , D27Y (50% C.38), D155H (2% C.36) | W131C (47%) | Q57H (76% 20A), S195P (6.3% 20A), S171L (80.7 19B) |
